# Characteristics and outcomes of patients hospitalized with interstitial lung diseases in Spain, 2014 to 2015

**DOI:** 10.1097/MD.0000000000015779

**Published:** 2019-05-24

**Authors:** Fernando Pedraza-Serrano, Rodrigo Jiménez-García, Ana López-de-Andrés, Valentin Hernández-Barrera, Gema Sánchez-Muñoz, Luis Puente-Maestu, Javier de-Miguel-Díez

**Affiliations:** a Respiratory Department, Hospital General Universitario Gregorio Marañón, Facultad de Medicina, Universidad Complutense de Madrid (UCM), Instituto de Investigación Sanitaria Gregorio Marañón (IiSGM); b Preventive Medicine and Public Health Teaching and Research Unit, Department of Health Sciences, Universidad Rey Juan Carlos, Alcorcón, Madrid, Spain.

**Keywords:** comorbidities, in-hospital mortality, interstitial lung diseases, pulmonary hypertension

## Abstract

To assess characteristics and outcomes of patients hospitalized with interstitial lung diseases (ILD) and to analyze patient's comorbidities, procedures, and in-hospital outcomes.

We identified patients hospitalized with idiopathic pulmonary fibrosis and others ILD such as hypersensitivity pneumonitis, cryptogenic organizing pneumonia, lymphangioleiomyomatosis, pulmonary Langerhans cell histiocytosis, and sarcoidosis in Spain during 2014 and 2015.

We identified 14,565 discharges among patients admitted for ILD in Spain during the study period: idiopathic pulmonary fibrosis (IPF) in 42.32% (n = 6164), sarcoidosis in 37.65% (n = 5484), hypersensitivity pneumonitis in 10.55% (n = 1538), cryptogenic organizing pneumonia in 7.06% (n = 1028), pulmonary Langerhans cell histiocytosis in 1.48% (n = 215), and lymphangioleiomyomatosis in 0.94% (n = 136). The most common associated comorbidities according to those included in the Charlson Comorbidity Index (CCI) were COPD, diabetes, and congestive heart disease. The presence of pulmonary hypertension increased the probability of dying in patients with idiopathic pulmonary fibrosis (OR 1.36; 95%CI 1.06–1.73). Patients with cryptogenic organizing pneumonia had the longest length of hospital stay and the highest percentage of hospital readmissions (23.64%). The highest IHM corresponded to the idiopathic pulmonary fibrosis (14.94%). Computed tomography of the chest was the procedure more used during admissions for ILD.

IPF was responsible for larger percentage of hospital admission among ILD in our study. In addition, the IHM were higher in IPF patients in comparison with those with other ILD. The most common associated comorbidity in ILD according to those included in the CCI was COPD. Computed tomography of the chest was the procedure more frequently used.

## Abstract 2

Supplemental Digital Content is available in the text

## 1 Introduction

The interstitial lung diseases (ILD) are a heterogeneous group of more than 200 acute and chronic disorders characterized by different degrees of pulmonary inflammation and fibrosis. Despite that several factors, including occupational or environmental agents, infections, certain drugs, radiation, and genetic predisposition, can be involved in their pathogenesis, the majority of the cases are idiopathic.^[1–3]^

There are few and sparse epidemiological data of ILD in Spain,^[4,5]^ but not only in this Mediterranean country, we can find the same problem in other countries of Europe,^[6–9]^ America^[10]^ and in the rest of the world.^[11]^ Moreover, changes in the classification of ILDs, in the diagnostic criteria, and in the definition of the different diseases, make the comparison of data very difficult.^[12]^ In addition, the epidemiological studies are based on different data collection system and heterogeneous methods of investigation, which make even more complex to obtaining accurate data.

International studies comparing hospitalizations and outcomes for ILD patients could provide more information of national patterns and help in the planning of healthcare. Discharge databases represent an excellent instrument that allows to perform a national epidemiology study of hospitalizations for ILD. In a previous study we have provided robust data about epidemiology of hospital admissions for idiopathic pulmonary fibrosis, but there is a lack of data on hospitalizations for other ILD in our country.^[13]^

Using the Spanish National Hospital Discharge Database (SNHDD), we aim to describe characteristics and outcomes of patients hospitalized with ILD diseases in Spain in years 2014 and 2015. In particular we analyzed patient's comorbidities, procedures, and in-hospital outcomes, such as in-hospital mortality (IHM), length of hospital stay (LOHS) and costs.

## 2 Methods

A retrospective, descriptive, epidemiological study was conducted using the SNHDD which compiles all public hospital data and therefore covers more than 95% of hospital discharges. The SNHDD includes patient variables (sex, date of birth), admission, and discharge dates, up to 14 discharge diagnoses, and up to 20 procedures performed during the hospital stay.^[14]^ We analyzed data collected between January 1, 2014 and December 31, 2015. We excluded those patients with missing data on age, sex, and those without precise information on their discharge status (alive or died during the hospitalization).

We selected all admissions for patients whose medical diagnosis include ILD as a primary or secondary diagnosis, coded as idiopathic pulmonary fibrosis (516.31), hypersensitivity pneumonitis (495.9), cryptogenic organizing pneumonia (516.36), lymphangioleiomyomatosis (516.4), pulmonary Langerhans cell histiocytosis (516.5), and sarcoidosis (135) in any diagnostic field according to the International Classification of Diseases, Ninth Revision, Clinical Modification (ICD-9-CM). We collected data from 2014 because previous to this year the ICD-9-CM codes for the ILD were not used in the SNHDD.

Clinical characteristics included information on overall comorbidity at the time of discharge, which was assessed by calculating the Charlson comorbidity index (CCI).^[15]^ We divided patients into 3 categories: CCI 0, as in those patients with no previously recorded disease; CCI 1, patients with 1 disease category and CCI ≥ 2, patients with 2 or more disease categories.

We retrieved data concerning all the specific comorbidities included in the CCI (acute myocardial infarction, congestive heart failure, peripheral vascular disease, cerebrovascular disease, dementia, chronic obstructive pulmonary disease (COPD), rheumatoid disease, peptic ulcer, mild liver disease, diabetes, hemiplegia/paraplegia, renal disease, cancer, moderate/sever liver disease, metastatic cancer, and AIDS) as described by Quan et al^[16]^ and that were applied to ICD-9-CM. Also, we retrieved data of pulmonary hypertension (ICD-9-CM codes 416.0 and 416.8).

We specifically identified the following procedures using the corresponding ICD-9-CM codes: heart echocardiogram (88.72), respiratory function test (89.37), ultrasound of lower limbs (88.77), lung scintigraphy (92.15), computed tomography of the chest (87.41), right heart catheterization (37.21), pulmonary arteriography (88.43), and lung biopsy (33.20, 33.24, 33.26, 33.27, 33.28).

We also analyzed the use of ventilatory support during admissions for interstitial lung disease. Use of non-invasive ventilation and invasive mechanical ventilation was determined based on procedure codes 93.90 and 96.04, respectively. We also identified the percentage of patients undergoing lung transplant (codes 33.50, 33.51, 33.52, and 33.56).

We estimated the readmissions rate (patients that had been discharged from the same hospital within the previous 30 days), the mean LOHS, and IHM. IHM was defined by the proportion of patients who died during admission for each year of study.

We also estimated costs. Costs were calculated using Diagnosis-Related Groups (DRG).^[17]^

### 2.1 Statistical analysis

A descriptive statistical analysis was performed for all continuous variables and categories. Variables are expressed as proportions as means with standard deviations or as medians with IQR.

Multivariable analysis of each ILD-associated IHM was conducted using logistic regression models with adjustment for age, sex, and other co-variables as appropriate.

To conduct the multivariable logistic regression models we first performed a univariable analysis of each variable, from which we selected variables for the multivariable analysis. We included all the variables whose univariable test result was significant and those we considered scientifically relevant according to the references reviewed. After fitting the multivariable model, we verified the importance of each variable included, by examining the Wald statistic for each variable and comparing each estimated coefficient with the coefficient from the univariable model containing only that variable. Variables that did not contribute to the model based on these criteria were eliminated, and a new model was fitted. The new model was compared with the old model using the likelihood ratio (LR) test. Furthermore, estimated coefficients for the remaining variables were compared to those from the full model. This process of deleting, refitting, and verifying continued until all the major variables were included in the model. Once the model was obtained, we looked more closely at the variables included (linearity) and checked for interactions.

All statistical analyses were performed with Stata version 10.1 (Stata, College Station, Texas, USA). Statistical significance was set at *P* < .05 (2-tailed).

### 2.2 Ethical aspects

Data confidentiality was maintained at all times as patient identifiers were deleted before the database was provided to the authors in order to maintain patient anonymity. It is not possible to identify patients on an individual level, either in this article or in the database. Given the anonymous and mandatory nature of the dataset, it was not necessary to obtain informed consent or approval by the ethics committee, in accordance with the Spanish legislation.

## 3 Results

The flowchart of study subject's selection is shown in Supplementary Figure 1. We identified 14,565 discharges among patients admitted for ILD in Spain in years 2014 and 2015. In 37.65% of cases (n = 5484), ILD was sarcoidosis, in 10.55% (n = 1538) was hypersensitivity pneumonitis, in 42.32% (n = 6164) was idiopathic pulmonary fibrosis, in 7.06% (n = 1028) was cryptogenic organizing pneumonia, in 1.48% (n = 215) was pulmonary Langerhans cell histiocytosis and in 0.94% (n = 136) was lymphangioleiomyomatosis

Table 1 shows sociodemographic and clinical characteristics and in-hospital outcomes of patients with interstitial lung diseases.

**Table 1:**
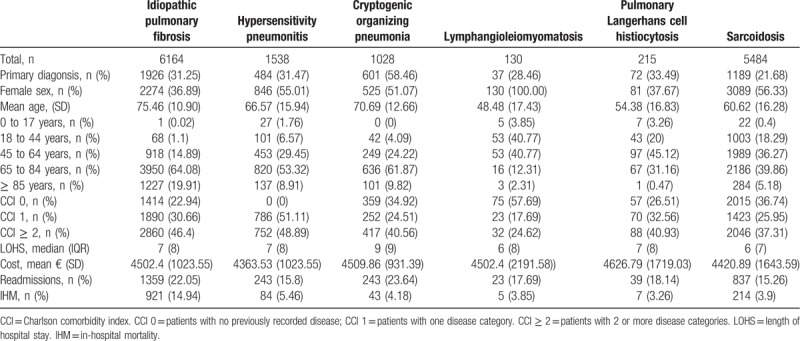
Hospitalizations with interstitial lung diseases as primary or secondary diagnosis in Spain (2014-2015).

We found that around 66% to 72% of cases, ILD were coded as a secondary diagnosis, except cryptogenic organizing pneumonia (41.54% of cases). The most common primary diagnoses in patients discharged with a secondary diagnosis of ILD were other specific respiratory disorders (idiopatic pulmonary fibrosis [IPF], 12.41%; hypersensitive pneumonitis, 12.53%; pulmonary Langerhans cell histiocytosis, 5.59%; cryptogenic organizing pneumonia, 5.15%; and sarcoidosis, 4.59%), pneumonia (lymphangioleiomyomatosis, 13.27%; cryptogenic organizing pneumonia, 11.48%; idiopatic pulmonary fibrosis, 8.85%; hypersensitive pneumonitis, 7.40%, and sarcoidosis, 3.80%) and acute and chronic respiratory failure (idiopatic pulmonary fibrosis, 9.41%; hypersensitive pneumonitis, 8.25%; pulmonary Langerhans cell histiocytosis, 6.29%, lymphangioleiomyomatosis, 4.08%; and sarcoidosis, 2.00%) (Supplementary Table 1).

Female sex hospitalizations was more frequent in all ILD highlighting lymphangioleiomyomatosis (100%), expect idiopathic pulmonary fibrosis, (36.89%) and pulmonary Langerhans cell histiocytosis (37.67%).

We found that patients with idiopathic pulmonary fibrosis, hypersensitivity pneumonitis, and cryptogenic organizing pneumonia had over 75, 66, and 70 years mean age respectively and many comorbidities according to the CCI. Patients with lymphangioleiomyomatosis had a median of 48.48 years (SD: 17.43) and had a lower values of CCI (57.69% without comorbidity). By last, patients with pulmonary Langerhans cell histiocytosis and sarcoidosis had a median of 54.38 years (SD 16.83) and 60.82 years (SD: 16.28) respectively and intermediate values of CCI.

Median LOHS for admissions for ILD was around 7 days, except in hospitalizations with cryptogenic organizing pneumonia (9 days) and lymphangioleiomyomatosis (6 days).

The mean cost per patient was around 4500€ in hospitalizations with idiopathic pulmonary fibrosis, cryptogenic organizing pneumonia, lymphangioleiomyomatosis, and pulmonary Langerhans cell histiocytosis. However, in patients with hypersensitivity pneumonitis and sarcoidosis was lower, around 4300€ and 4400€ respectively.

Proportion of readmissions varied between 14.31% in patients with sarcoidosis and 23.64% in patients with cryptogenic organizing pneumonia.

We found that IHM in patients with idiopathic pulmonary fibrosis was 14.94%. In the rest of the ILD was 5.46% in patients with hypersensitivity pneumonitis, 4.18% with cryptogenic organizing pneumonia, 3.90% with sarcoidosis, 3.68% with lymphangioleiomyomatosis and 3.26% with pulmonary Langerhans cell histiocytosis.

The most common associated comorbidity for patients hospitalized with ILD in Spain in years 2014 and 2015 according to the CCI was COPD, as can been seen in Table 2. Other comorbidities were diabetes not complicated and congestive heart disease (24.94% and 21.03% in patients with idiopathic pulmonary fibrosis, 21.2% and 14.89% with hypersensitivity pneumonitis, 22.37% and 17.02% with cryptogenic organizing pneumonia and 20.39% and 13.38% with sarcoidosis, respectively).

**Table 2:**
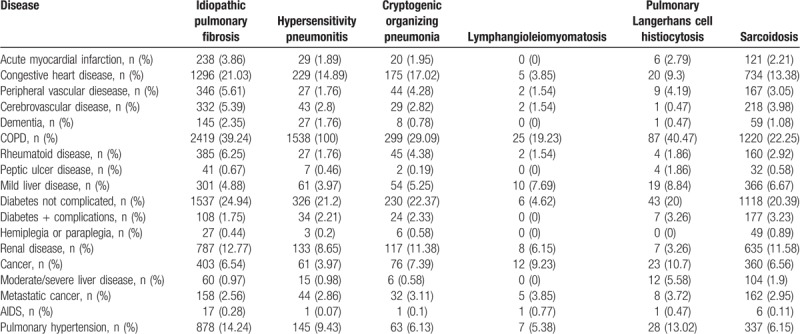
Associated comorbidities included in the Charlson Comorbidity Index and Pulmonary Hypertension for patients hospitalized with interstitial lung diseases as primary or secondary diagnosis in Spain (2014–2015).

Table 3 shows the diagnostic procedures and treatments for patients hospitalized with ILD. Computed tomography of the chest was the procedure more used in patients with interstitial lung diseases. Other procedures were respiratory function tests (5.47% in patients with idiopathic pulmonary fibrosis, 8.19% with hypersensitivity pneumonitis, 7.39% with cryptogenic organizing pneumonia, 8.84% with pulmonary Langerhans cell histiocytosis and 4.36% with sarcoidosis) and non-invasive mechanical ventilation (5.3% in patients with idiopathic pulmonary fibrosis, 5.14% with hypersensitivity pneumonitis, 3.7% with cryptogenic organizing pneumonia, 2.94% with lymphangioleiomyomatosis, 5.12% with pulmonary Langerhans cell histiocytosis and 2.1% with sarcoidosis). Regarding lung biopsy it was conducted in 7.32% of patients with Idiopathic pulmonary fibrosis, 17.04% for those with hypersensitivity pneumonitis, 27.63% with cryptogenic organizing pneumonia, 12.50% with lymphangioleiomyomatosis, 19.53% with pulmonary Langerhans cell histiocytosis, and 8.37% with sarcoidosis.

**Table 3:**
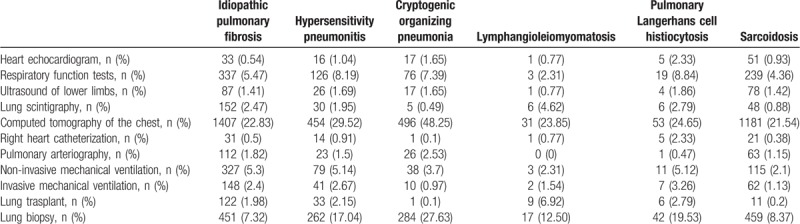
Diagnosis procedures and treatments for patients hospitalized with interstitial lung diseases as primary or secondary diagnosis in Spain (2014–2015).

After controlling for possible confounders using logistic regression models, we found that the risk of IHM in patients with idiopathic pulmonary fibrosis, cryptogenic organizing pneumonia, and pulmonary Langerhans cell histiocytosis was higher in older ages (OR 1.02; 95%CI1.01–1.03, OR 1.04; 95%CI1.02–1.06 ,and OR 1.06; 95%CI 1.05–1.07, respectively.

Table 4 summarizes the results of the multivariable analysis of factors associated with IHM among patients hospitalized with idiopathic pulmonary fibrosis, hypersensitivity pneumonitis, and sarcoidosis. The factors associated with IHM included age, peripheral vascular disease, cerebrovascular disease, dementia, mild lever disease, cancer including metastatic cancer, and pulmonary hypertension.

**Table 4:**
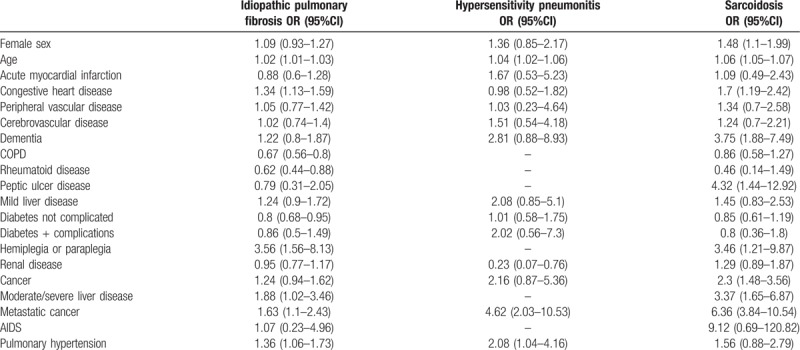
Multivariable analysis of factors associated with in-hospital deaths among patients hospitalized with interstitial lung diseases in Spain (2014–2015).

The presence of pulmonary hypertension increased the probability of dying in patients with idiopathic pulmonary fibrosis (OR 1.36; 95%CI 1.06–1.73) and with hypersensitivity pneumonitis (OR 1.92; 95%CI 1.28–2.87), but not in those with sarcoidosis (OR 1.56; 95%CI 0.88–2.79).

In patients with sarcoidosis the risk of IHM was higher in females (OR 1.48; 95%CI 1.1–1.899). In addition, the presence of congestive heart failure, dementia, peptic ulcer disease, hemiplegia or paraplegia, and cancer including metastatic cancer, also increased the risk of in hospital mortality in these patients. The presence of moderate/severe liver disease in patients with idiopathic pulmonary fibrosis and with sarcoidosis increased the probability of died (OR 1.88; 95%CI 1.02–3.46, and OR 3.37; 95%CI 1.65–6.87). However, the presence of COPD, rheumatoid disease, and diabetes not complicated in patients with idiopathic pulmonary fibrosis were associated with lower mortality (OR 0.67; 95%CI 0.56–0.80, OR 0.62; 95%CI 0.44–0.88, and OR 0.8; 95%CI 0.68–0.95). Furthermore, the presence of renal disease in patients with hypersensivity pneumonitis was also associated with lower mortality (OR 0.23; 95%CI 0.07–0.76).

## 4 Discussion

In our study, the most frequent ILD were idiopathic pulmonary fibrosis (42.32%) and sarcoidosis (37.65%) as in most previous published studies.^[4,18,19]^ However, other authors have reported sarcoidosis as the most common condition.^[6,7]^ On the other hand, in an important Danish series the most frequent ILD was idiopathic pulmonary fibrosis, followed by connective tissue disease-related ILD, but this authors excluded all cases of sarcoidosis.^[20]^

The most prevalent associated comorbidity in our study was COPD, possibly related to emphysema. Since smoking is a risk factor for developing both, COPD and IPF, it is common that the 2 entities occurs in the same patient either as different diseases or as the syndrome of combined pulmonary fibrosis and emphysema (CPFE).^[21]^ The described prevalence of this syndrome is very variable, depending on the series: Otsuka et al^[22]^ described, in their cohort of 831 patients with lung cancer and surgical resection, only 23 patients (2.8%) with this diagnosis. On the other hand, Ryerson et al^[23]^ found CPEF in 8% of patients with idiopathic pulmonary fibrosis. However, the prevalence of emphysema in the idiopathic pulmonary fibrosis sample of Ye et al^[24]^ was 56%. In our study, we found a prevalence of 39%, in the average of the series reviewed. This wide difference between cohorts can be due to several causes such as heterogeneous group of patients, inaccurate definition of this syndrome and different radiological criteria for quantifying the emphysema.

In smokers, COPD may also coexist with other ILD such as pulmonary Langerhans cell histiocytosis.^[25]^ However, it is often difficult to differentiate between emphysema and cyst in the upper lobes (radiological characteristic of pulmonary Langerhans cell histiocytosis) or even between emphysema and honeycomb by expert radiologists. Also, emphysema has been associated with several other non smoking-related ILD like hypersensitivity pneumonitis^[26]^ and even in connective tissue disease like systemic sclerosis.^[27]^

With regard to diabetes, we found that all the ILD analyzed in our study, except lymphangioleiomyomatosis, presented this comorbidity with a high frequency. It could be due that most of these diseases are treated with systemic corticosteroids, which is known to alter glucose levels in blood, especially during hospital admissions. Similar result have been reported by other authors in different countries and very different ethnic populations, such as Enomoto et al^[28]^ in Japan, Gribbin et al^[29]^ in United Kingdom, Hyldgaard et al^[30]^ in Denmark and García-Sancho Figueroa et al^[31]^ in Mexico. But it seems that the presence of diabetes mellitus is not only due to the use of corticosteroids therapy, since these findings persisted after exclusion of individuals treated with them.

Pulmonary hypertension associated with idiopathic pulmonary fibrosis is usually mild; only in 10% of the patients is more severe than would be expected.^[32]^ Data about prevalence of pulmonary hypertension-idiopathic pulmonary fibrosis are very variable, from 3% to 86%.^[33,34]^ We have found a value of 14.24% in our study. This wide range may be due to different diagnosis methods used, to heterogeneous population groups studied or even to the differences in the severity of disease. It is logical to think that the presence of pulmonary hypertension worsen the prognosis of idiopathic pulmonary fibrosis. In fact, most studies show this finding.^[33,35,36]^ By contrast Oda et al,^[37]^ in a recent study in Japan similar in design to ours, did not observe that pulmonary hypertension influenced the prognosis of hospitalized patients with idiopathic pulmonary fibrosis.

After idiopathic pulmonary fibrosis, pulmonary Langerhans cell histiocytosis was the ILD most frequently accompanied by pulmonary hypertension in our study. Previous studies have also reported that pulmonary hypertension associated to pulmonary Langerhans cell histiocytosis was more frequent and severe than in other ILD. Farkouth et al^[38]^ indicated that it might be due to an intrinsic pulmonary vascular disease, in which the pulmonary circulation is involved independently of lung parenchyma and small airway.

Sarcoidosis can also be associated with pulmonary hypertension. The presence of this complication worsens the prognosis. The prevalence of pulmonary hypertension found in our study in patients with sarcoidosis was 6.1%, being in the range of the prevalence described by other authors, which is between 5% and 15%.^[35]^

Regarding the diagnostic procedures, computed tomography of the chest was the test most frequently performed during hospital admissions, especially in patients with cryptogenic organizing pneumonia. It could be due that cryptogenic organizing pneumonia is almost an exclusion diagnosis, so it is very frequent that it presents as a bacterial pneumonia that does not respond to antibiotics. Thereby, in these “special pneumonias” a computed tomography of the chest is almost mandatory to get closer to an accurate diagnosis. Others diagnosis procedures, as respiratory function test, are usually developed in outpatients when they are in their baseline situation, and not during hospital admission. The same could have happened with the echocardiogram and the right heart catheterization, which could justify the scarce percentage found in the use of these procedures in our study.

As for LOHS, the longest corresponded to cryptogenic organizing pneumonia (9 days) in comparison with the average (7 days). This could be explained by the same reason; the diagnosis of this disease is usually delayed until a computed tomography of the chest is performed, after waiting for the response of the antibiotics, making the hospital stay last longer than usual.^[39]^ This could also be the reason for the high cost of this disorder. However, pulmonary Langerhans cell histiocytosis was the ILD associated with a highest cost, being the high rate of pneumothorax one of the reason that could contribute to explain this finding. In fact, this complication is one of the main reasons for hospital admission in these patients, and it increases the costs.^[40]^

Cryptogenic organizing pneumonia was associated with the highest rate of readmissions. This could be because in cryptogenic organizing pneumonia recurrences are very frequent. Although sarcoidosis should have a high rate of readmissions for the same reason, we found the lowest rate in this case. It is possible that most of sarcoidosis recurrences would be treated like outpatients.

Regarding mortality, IHM was higher in idiopathic pulmonary fibrosis patients than in the rest of the ILD studied in our study. By contrast, Oda et al^[37]^ found a higher mortality in acute interstitial pneumonia. They also found that lung cancer and bacterial pneumonia influenced on IHM in patients with idiopathic pulmonary fibrosis. In a recent Korean study, based on ICD codes like ours, Choi et al^[41]^ also observed that patients with ILD have a significantly higher risk of death than matched controls.

Our study has some strengths and limitations. The most important strengths are its standardized methodology applied and especially its large sample size. However, the current study has some limitations that we have to take into account when interpreting our results. The potential source of bias comes from the use of ICD-9-CM. The main concern of using administrative database is to get an accurate diagnosis. Moreover, in our data we do not known if diagnoses were performed by pneumologists or if they were based on multidisciplinary discussions in all cases. On the other hand, it is possible that we could underestimated the incidence of ILD, because of inaccurate data especially in asymptomatic patients with ILD, Also, ILD outcomes should be affected by treatment, a variable that we could not include in our study. Nevertheless previous studies using database data in USA^[42]^ and in Asia^[41]^ reported similar results. Although SNHDD have some limitations, it has a great advantage that it is mandated by the National Public Health System, and it includes almost 100% of admission in Spain and it is periodically audited.

Finally, we did not include years 2016 and 2017 in our investigation because from year 2016 the hospital discharge data in Spain is being codified according to the “International Classification of Diseases-10 review-clinical modification” and not according to the “International Classification of Diseases-9 revision-modification clinic” as in our study period. The Spanish Ministry of Health has informed us that the first years after the change from ICD9 to ICD10 the data may not be as reliable and extensive as it was for ICD9.

In conclusion, both, the incidence of hospitalizations for IPF and the IHM were higher in patients with idiopathic pulmonary fibrosis, in comparison with other ILD in our study. The presence of pulmonary hypertension increased the probability of dying among IPF theses patients. These results highlight the need for a careful diagnosis and treatment of these heterogeneous group of diseases. So, nowadays the multidisciplinary discussion is the gold-standard for achieve an accuracy diagnosis. The new anti-fibrotic drugs and their increasingly widespread use could change these results in the future.

## Acknowledgments

We would like to thank the Spanish Ministry of Health and Social Policy for providing the records of the Spanish National Hospital Discharge Database.

## Author contributions

**Conceptualization:** Fernando Pedraza-Serrano, Rodrigo Jiménez-García, Javier de Miguel-Diez.

**Formal analysis:** Valentin Hernandez-Barrera.

**Investigation:** Javier de Miguel-Diez.

**Methodology:** Fernando Pedraza-Serrano, Rodrigo Jiménez-García, Valentin Hernandez-Barrera, Gema Sanchez-Muñoz, Luis Puente-Maetsu.

**Supervision:** Rodrigo Jiménez-García, Ana Lopez-de-Andres, Javier de Miguel-Diez.

**Writing – original draft:** Fernando Pedraza-Serrano, Gema Sanchez-Muñoz.

**Writing – review & editing:** Rodrigo Jiménez-García, Ana Lopez-de-Andres, Luis Puente-Maetsu, Javier de Miguel-Diez.

## Supplementary Material

Supplemental Digital Content
