# Supplementary material for: Characteristics and outcomes of patients hospitalized with interstitial lung diseases in Spain, 2014 to 2015
Source: Medicine (Baltimore). 2019 May 24;98(21):e15779. doi: 10.1097/MD.0000000000015779 (PMC6571208; doi:10.1097/MD.0000000000015779)
Supplement: Supplemental Digital Content [file medi-98-e15779-s001.pdf]

Supplementary Table 1. Most common primary diagnosis in patients discharged with a secondary diagnosis of interstitial lung diseases.

	Diagnosis	N	%
**Idiopathic pulmonary fibrosis**	Other specified respiratory disorders	526	12.41
	Acute and chronic respiratory failure	399	9.41
	Pneumonia	375	8.85
	Congestive heart failure	255	6.02
**Hypersensitivity pneumonitis**	Other specified respiratory disorders	132	12.52
	Acute and chronic respiratory failure	87	8.25
	Pneumonia	78	7.40
	Acute respiratory failure	66	6.26
**Cryptogenic organizing pneumonia**	Pneumonia	49	11.48
	Congestive heart failure	36	8.43
	Acute respiratory failure	33	7.73
	Other specified respiratory disorders	22	5.15
**Lymphangioleiomyomatosis**	Pneumonia	13	13.27
	Other pneumothorax	8	8.16
	Acute and chronic respiratory failure	4	4.08
	Benign neoplasm of unspecified kidney	3	3.06
**Pulmonary Langerhans cell histiocytosis**	COPD with exacerbation	11	7.69
	Acute and chronic respiratory failure	9	6.29
	Acute bronchitis	8	5.59
	Other specified respiratory disorders	8	5.59
**Sarcoidosis**	Other specified respiratory disorders	197	4.59
	Pneumonia	163	3.80
	Congestive heart failure	156	3.63
	Acute and chronic respiratory failure	86	2.00

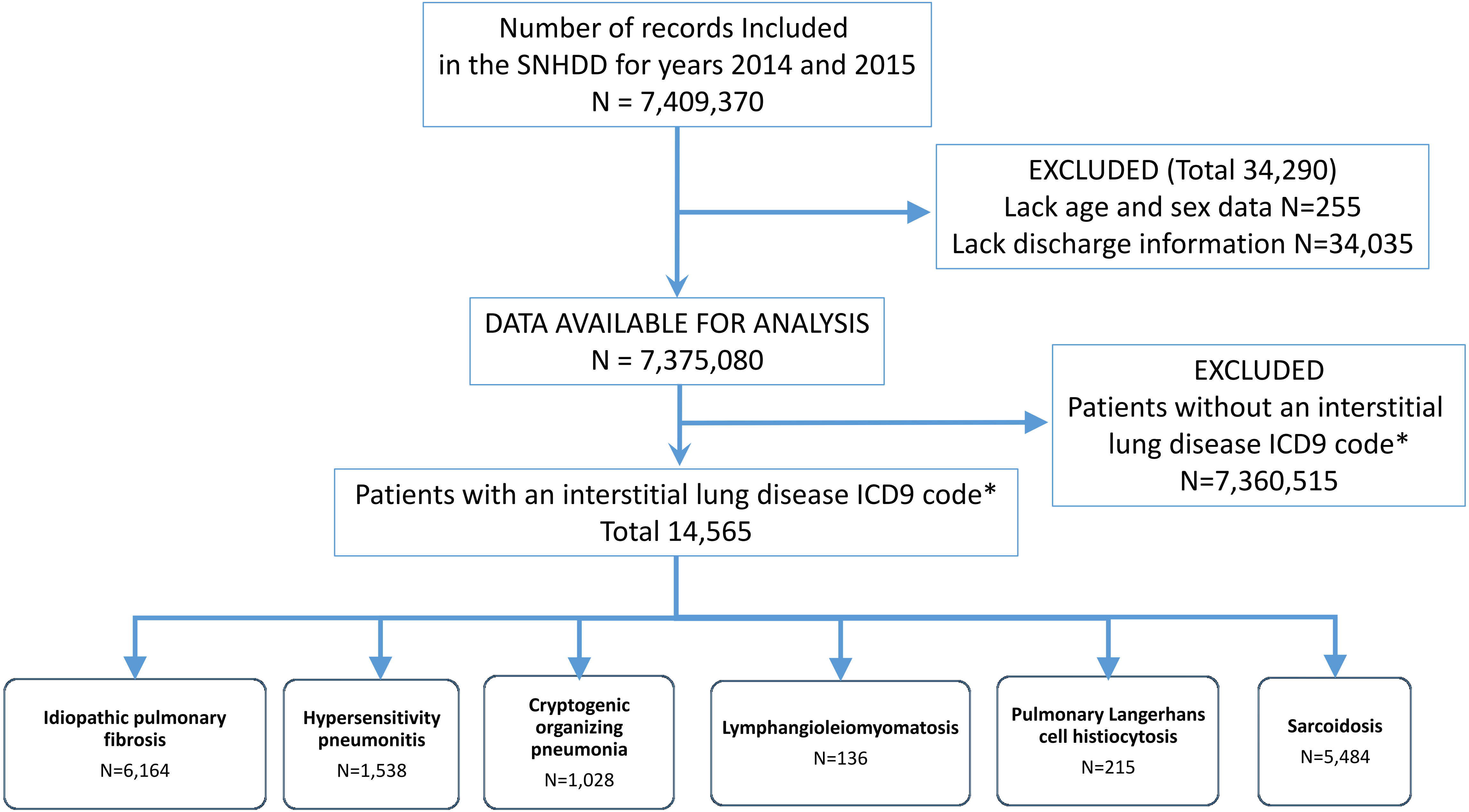

SNHDD : Spanish National Hospital Discharge Database * 516.31, 495.9, 516.36, 516.4, 516.5, 135
